# TRIM41 contributes to the pathogenesis of airway allergy by compromising dendritic cells’ tolerogenic properties

**DOI:** 10.1016/j.isci.2024.110067

**Published:** 2024-05-21

**Authors:** Qiuying Peng, Xiangqian Luo, Lihua Mo, Xuejie Xu, Yu Liu, Dabo Liu, Pingchang Yang

**Affiliations:** 1 Department of Pediatric Otolaryngology, Shenzhen Hospital of Southern Medical University, Shenzhen, China; 2 Department of Pediatrics, Guangzhou Panyu Maternal and Children Health Hospital, Guangzhou, China; 3 Institute of Allergy & Immunology of Shenzhen University and State Key Laboratory of Respiratory Diseases Allergy Division at Shenzhen University, Shenzhen, China; 4 Department of General Practice Medicine, Third Affiliated Hospital of Shenzhen University, Shenzhen, China

**Keywords:** Molecular biology, Immunology, Components of the immune system

## Abstract

Dendritic cells (DC) play a crucial role in the initiation of immune responses. TRIM41, an E3 ubiquitin ligase, can facilitate targeting protein degradation. The purpose of this study is to analyze the role of TRIM41 in the pathogenesis of airway allergy (AA) and the impact of regulating TRIM41 on suppressing AA. We observed that the airway DCs of AA mice had a higher expression of *Trim41*. The expression of *Trim41* in airway DCs was associated with the DCs’ tolerogenic functions of AA mice. The AA responses, including increased amounts of eosinophil peroxidase, mast cell protease-1, Th2 cytokines, and specific IgE in bronchoalveolar lavage fluids, were positively correlated with the *Trim41* expression in mouse airway DCs. TRIM41 induced c-Maf degradation and interfered with the *Il10* expression in airway DCs, which could be counteracted by inhibiting TRIM41. Regulation of TRIM41 mitigated experimental AA responses.

## Highlights

• High Trim41 amount in airway dendritic cells (DCs) of airway allergy (AA) mice
• *Trim41* compromises DC’s tolerogenic functions of AA mice
• Regulation of Trim41 restores DC’s immune tolerogenic ability
• Suppression of TRIM41 mitigates experimental AA responses

## Graphical abstract

**Figure:**
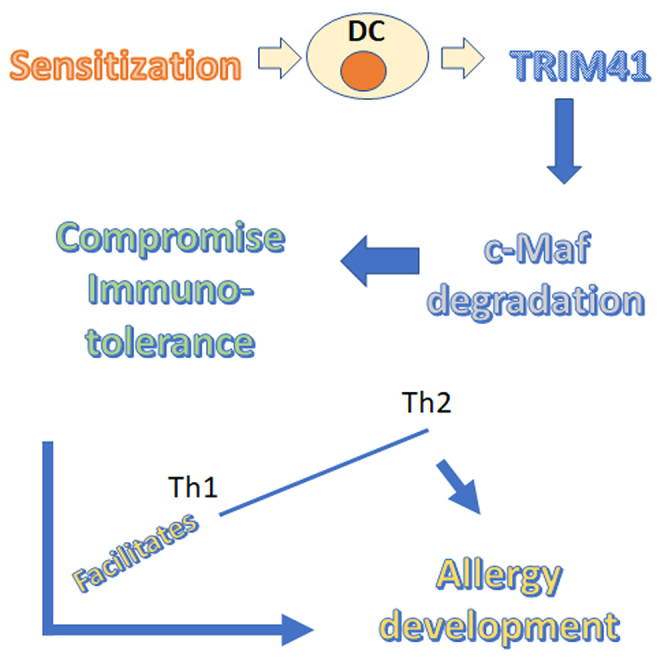

## Introduction

Airway allergy is an adverse response by the immune system in the airways to harmless airborne antigens. Airway allergy mainly includes allergic rhinitis and asthma. Clinical signs of airway allergy include paroxysmal attacks, profound secretions, and difficulty in breathing.^1^ In the early stages, the attacks of airway allergy are self-limited. It may last for a long time in the advanced stages due to the fibrosis in the airway mucosa.^2^ Complications may occur if airway allergy is not managed properly, such as chronic rhinitis, chronic rhinosinusitis, emphysema, chronic obstructive pulmonary diseases, or even pulmonary-cardiac diseases.^3^ Current therapies for airway allergy focus on the clinical symptom control. Antihistamine agents and steroids are commonly used in allergic clinics.^4^ In fact, the prevalence of airway allergy exceeds 20% in the world, which has kept rising over the last several decades.^5^ This reflects that the therapeutic remedies for airway allergy are not satisfactory and require further in-depth investigation.

It is widely agreed that Th2 polarization plays a crucial role in the pathogenesis of airway allergy. Th2 polarization indicates a condition in which Th2 cells aggregate beyond the local tissues. More than needed Th2 cytokines are produced by Th2 cells, which saturate the local tissues.^6^ Th2 cytokines drive B cells to differentiate into plasma cells and produce IgE antibodies.^6^ IgE causes mast cells to be sensitized. When re-exposed to specific antigens, mast cells become activated and release allergic mediators, such as histamine, tryptase, and serotonin, to initiate allergic reactions.^7^ Yet, the mechanism of Th2 polarization is not fully understood. Remedies used to correct Th2 polarization are limited, and they warrant further investigation.

The immune regulatory system in the body is responsible for tightly regulating immune responses in general. The canonical immune regulatory cells are regulatory T cells (Treg cells) and regulatory B cells (Breg cells).^8^^,^^9^ Interleukin (IL)-10 is an important mediator released by Breg cells and type 1 regulatory T cells (Tr1 cells), by which Breg cells and Tr1 cells suppress other immune cell responses. Immune regulation is responsible for limiting excessive immune responses to prevent self-injury.^10^ It has been recognized that the dysfunctional status of Breg cells and Tr1 cells in allergic diseases, including airway allergy.^11^ The underlying mechanism responsible for inducing immune regulatory cell dysfunction is not fully understood yet.

Excessive ubiquitination is one of the biochemical phenomena that can lead to the degradation of certain proteins. Factors inducing IL-10 reduction include speeding up RNA decay, hypermethylation of promoters, and excessive ubiquitination. We have identified that ubiquitin E3 ligases can regulate the immune regulatory cell functions.^12^^,^^13^^,^^14^^,^^15^ Here, we provide information on the tripartite motif (TRIM)41 family proteins, which play a critical role in regulating the expression of Il10 in airway DCs. Excessive expression of *Trim41* in DCs was associated with the impaired *Il10* expression, which could be improved by the presence of ANXA10. Experimental airway allergy was mitigated by the administration of ANXA10.

## Results

### High expression of *Trim41* in airway DCs of AA mice

**Figure 1:**
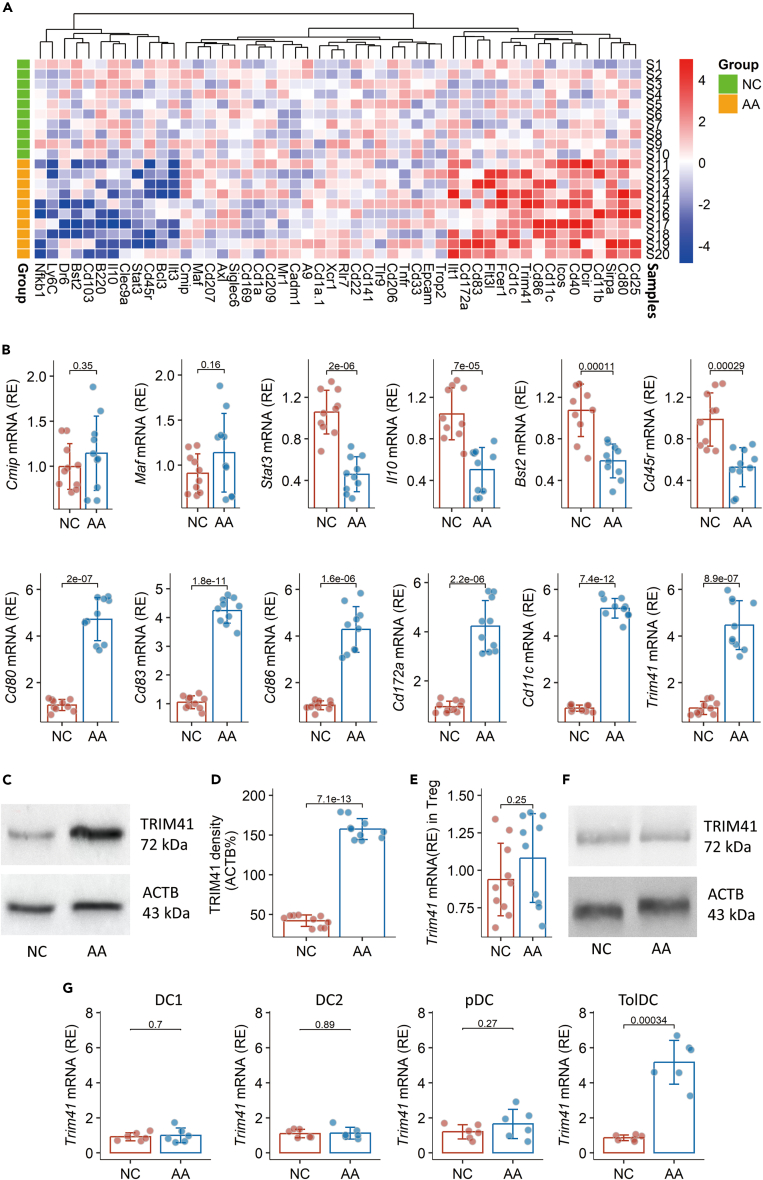
Gene profile of airway DCs DCs were isolated from the airway tissues of NC mice (*n* = 10) and AA mice (*n* = 10) and analyzed by RNA-seq and RT-qPCR. (A) A heatmap show the gene activity levels. (B) Mean ± SD of mRNA amounts of indicated molecules. (C and D) Immunoblots show TRIM41 protein in DCs. Bars show mean ± SD of TRIM41 densitometry results. (E) The *Trim41* mRNA levels in Tregs. (F) The protein levels of TRIM41 in Tregs. (G) Tthe amounts of *Trim41* mRNA in DC subtypes. Each dot in bars presents one sample. Statistics: Student’s t test. *p* values are presented in figures. Abbreviations: NC: naive control. AA: airway allergy. RNA-seq: RNA sequencing.

An AA mouse model was established (Figure S1 in supplemental materials). Dendritic cells (DCs) were purified (the DC gating strategy is presented in Figure S2) from the mouse airway tissues to be analyzed by RNA sequencing (RNA-seq). In total of 11,565 genes were analyzed, in which 366 genes were upregulated and 289 genes were downregulated. The Trim41 gene activity was higher in the top 50 differentially expressed genes (DEGs). There was a marked decrease in the expression of the *Il10* gene and plasmacytoid DC-related genes. It should be noted that the expression of *Cmip* and *Maf* in DCs did not decrease in the AA group. The genes related to type 2 CD (DC2) and costimulatory factors were significantly upregulated (Figure 1A). The DEGs of interest in DCs were verified by conventional RT-qPCR (Figure 1B). Western blotting was used to confirm the elevated expression of TRIM41 (Figures 1C and 1D). The *Trim41* expression in DCs was negatively correlated with the AA responses and the immune tolerogenic functions of DCs (Figure S3). A positive correlation was detected between *Trim41* expression in airway DCs and their tolerogenic functions (Figure S4). In addition, the expression of TRIM41 in Tregs was not significantly different between the NC group and AA group (Figures 1E and 1F). Furthermore, we found the amounts of *Trim41* mRNA mainly elevated in TolDCs among DC subtypes (Figure 1G). The results indicate that airway DCs from AA mice show elevated expression of *Trim41* and the impaired *Il10* expression status in DCs.

### Elevated *Trim41* expression in airway DCs is associated with DCs’ tolerogenic functions of mice with AA

**Figure 2:**
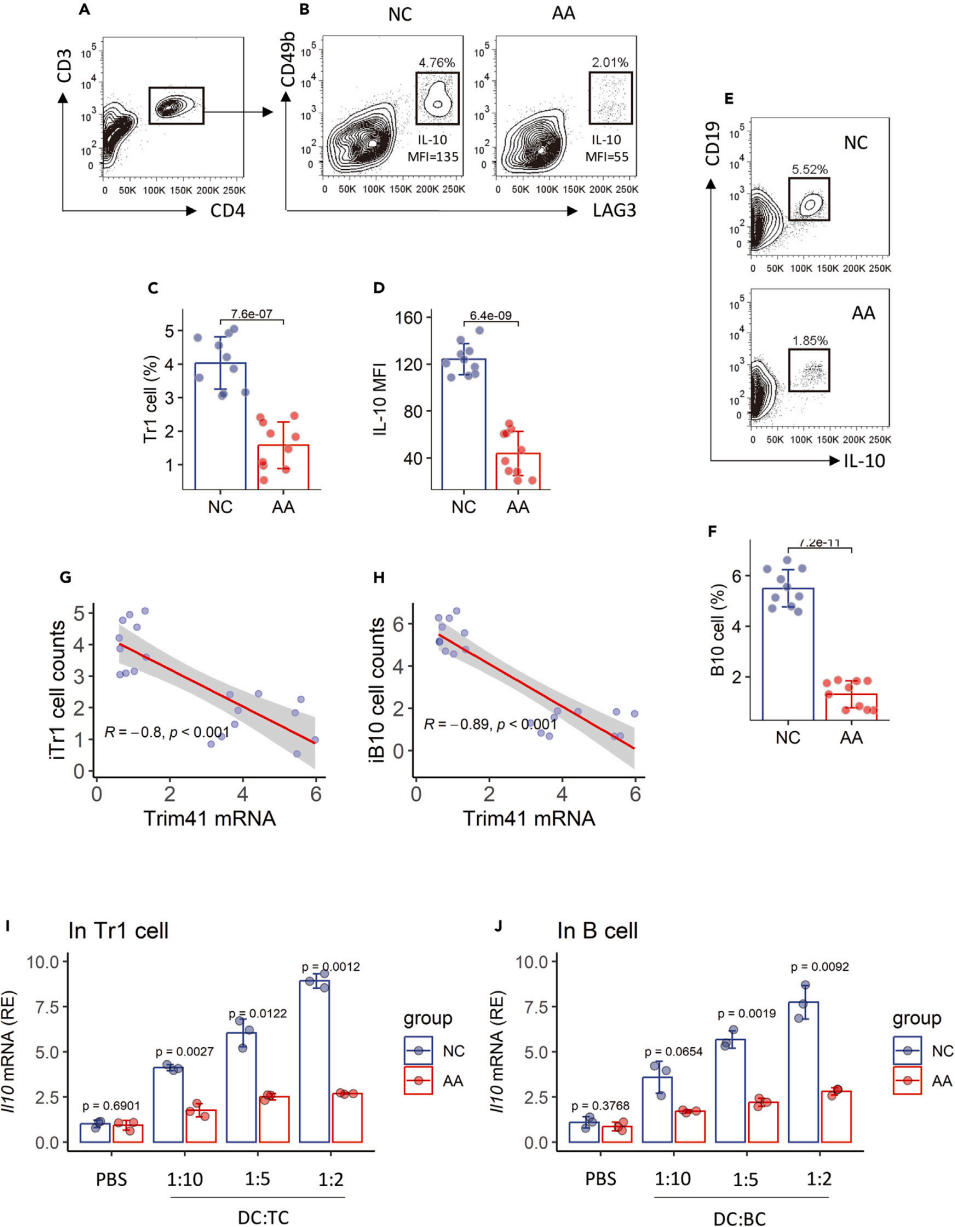
Assessment of immune tolerogenic functions of airway DCs of AA mice (A–F) AMCs were prepared with the airway tissues of NC mice (*n* = 10) and AA mice (*n* = 10). AMCs were analyzed by FCM. (A) CD3^+^CD4^+^ T cells were gated, from which Tr1 cells were gated (B). (C and D) Mean ± SD of Tr1 cell counts (C) and IL-10 MFI in Tr1 cells (D). (E) B10 cells were gated. (F) Mean ± SD of B10 cell counts. (G and H) Correlation between Trim41 mRNA (data are presented in Figure 1) and induced Tr1 cell (G) or induced B10 cell (H). (I and J) DCs were cocultured with naive CD4^+^ T cells (I) or naive B cells (J) for three days in the presence of PMA (50 ng/mL) and ionomycin (100 ng/mL). T cells and B cells were sorted, and analyzed by RT-qPCR. Bars show mean ± SD of *Il10* mRNA amounts from three independent experiments. Each dot in bars presents one sample. Statistics: Student’s t test (E, F, and H) or ANOVA + Bonferroni test (I and J). *p* values are presented in figures. Abbreviations: NC: naive control. AA: airway allergy. DC: dendritic cell. FCM: flow cytometry. PMA: pPhorbol myristate acetate. ViD: an amine reactive dead cell staining dye. iTr1 cell: induced Tr1 cell. iB10 cell: induced B10 cell.

The tolerogenic functional status of DCs in the airways of AA mice was assessed by counting the numbers of Tr1 cells and IL-10^+^ B cells (B10 cells) in the airway tissues. Airway mononuclear cells (AMCs) were isolated from the airway tissues of mice. Flow cytometry (FCM) was used to analyze AMCs. Significantly lower frequencies of Tr1 cells and B10 cells were found in AA mice compared to naive control (NC) mice. The IL-10 staining (presented by MFI, mean fluorescence intensity) was also weaker in Tr1 cells of AA mice than that in NC mice (Figures 2A–2F). A negative correlation was detected between the Tr1 cell frequency (Figure 2G) or the B10 cell frequency (Figure 2H) and the expression of *Trim41* in airway DCs.

Tr1 cells and B10 cells are the major components of the immune tolerant system.^16^ The results indicate that the immune tolerogenic system is compromised in AA mice. Both cell types can be induced by IL-10-producing cells, such as tolerogenic DCs.^17^^,^^18^ Thus, we next checked the tolerogenic ability of airway DCs. Total DCs (the gating strategy of DCs is presented in Figure S2) were purified from AMCs using FCM cell sorting. B220^+^ naive B cells and CD4^+^CD62L^+^ T cells were isolated from the spleen of naive mice. B cells or CD4^+^ T cells were cocultured with the DCs (in gradient numbers) in the presence of PMA and ionomycin (cell activators) for 3 days. Cells were analyzed by FCM. The induction of *Il10* expression in B cells and CD4^+^ T cells by DCs was regarded as an indicator of DCs’ immune tolerogenic functions. The results showed that DCs from naive control (NC) mice (the NC DCs) induced the *Il10* expression in naive B cells and naive CD4^+^ T cells in a DC number-dependent manner. The induced *Il10* mRNA amounts were markedly lower in B cells and CD4^+^ T cells by AA DCs as compared to that by NC DCs (Figures 2I and 2J). A negative correlation was detected between the *Trim41* expression in DCs and the DCs’ immune tolerogenic functions. The findings indicate that the immune tolerogenic abilities of airway DCs from AA mice are diminished.

### The expression of *Trim41* in airway DCs is linked to the AA responses

**Figure 3:**
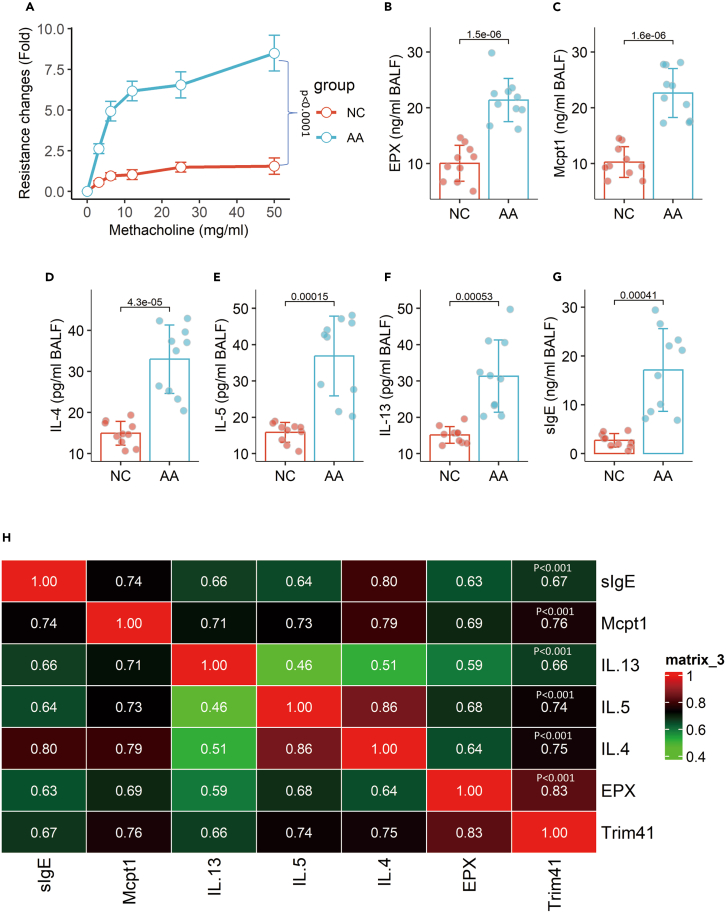
Association between DC’s *Trim41* expression and AA response (A) Mouse airway resistance changes in response to methacholine challenge. (B–G) A mean ± SD of the mounts of allergic mediators, Th2 cytokines and sIgE in BALF. (H) Correlation coefficients between DC’s *Trim41* expression and AA response. Each dot in bars presents one sample. Statistics: Student’s t test (B–G) and Pearson correlation coefficient test (A and H). *p* values are presented in figures. Each group consists of 10 mice. Abbreviations: NC: naive control. AA: airway allergy. sIgE: mite specific IgE. BALF: bronchoalveolar lavage fluid.

To assess the immune tolerogenic functions of DCs in AA, an AA mouse model was established (Figure S2). Elevated amounts of AA responses were detected in AA mice, which include airway resistance changes in response to the challenge with methacholine, the amounts of eosinophil peroxidase (EPX), mast cell protease-1 (Mcpt1), IL-4, IL-5, IL-13, mite specific IgE (sIgE), and sIgG1 in bronchoalveolar lavage fluids (BALF) (Figures 3A–3G). Notably, the AA responses were positively correlated with the expression of *Trim41* in airway DCs (Figure 3H). The results implicate a link between the expression of *Trim41* in airway DCs and the pathogenesis of AA.

### TRIM41 interferes with the IL-10 production in airway DCs

**Figure 4:**
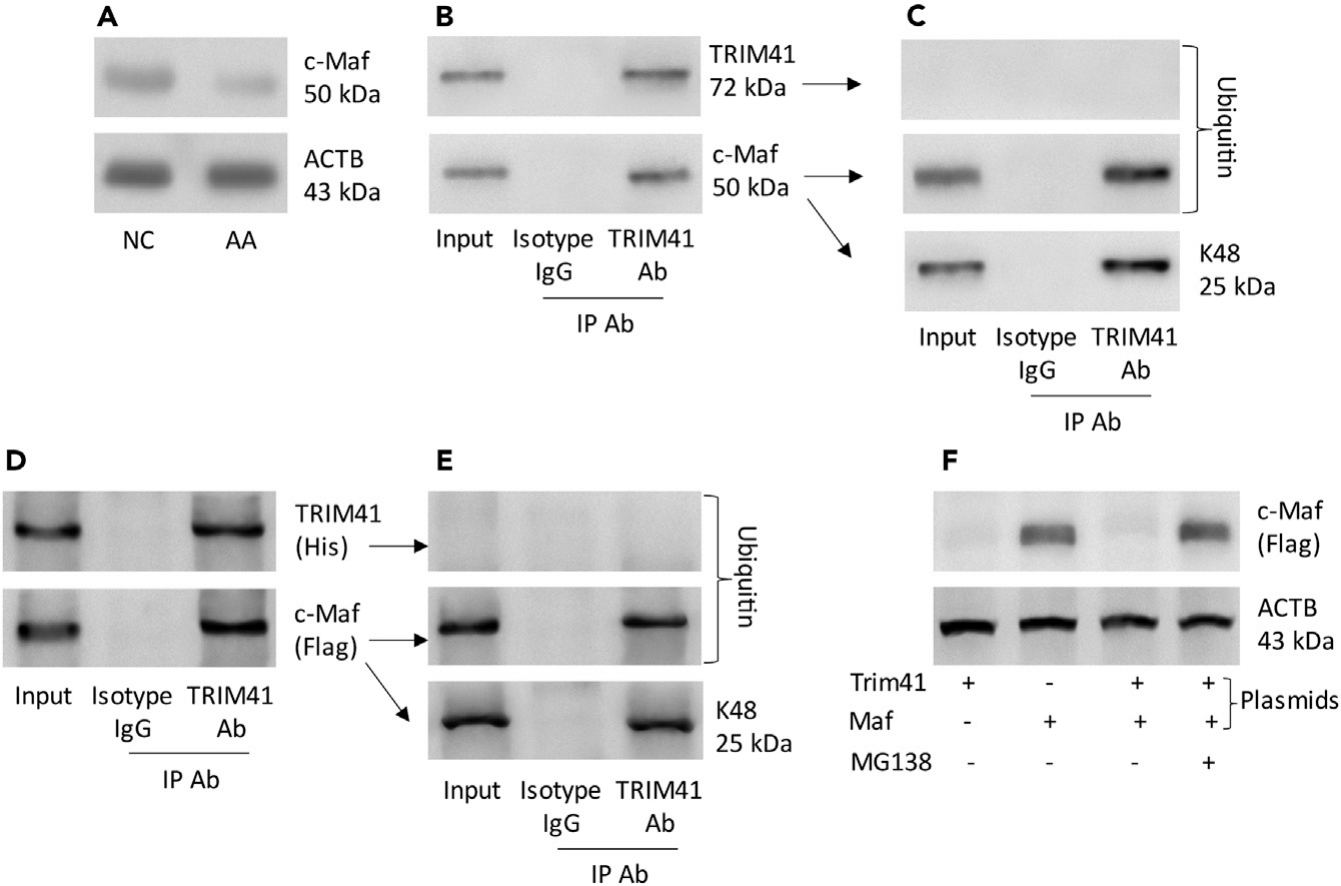
TRIM41 causes c-Maf protein degradation (A–C)Pprotein samples were extracted from DCs isolated from the airway tissues of AA mice. (A) Immunoblots show c-Maf protein abundance. (B) IP results show a complex of TRIM41 and c-Maf. (C) The PVDF membrane in (B) was treated with the “peeling-re-staining” approach and stained with a ubiquitin Ab. Immunoblots show the colocalization of c-Maf and ubiquitin as well as K48. (D) HEK293 cells were transfected with His-*Trim41*-plasmids and Flag-*Maf*-plasmids. The immunoblots show a complex of recombinant (r) TRIM41 and rc-Maf. (E) The PVDF membrane in (D) was treated with the “peeling-re-staining” approachnd stained with a ubiquitin Ab. Immunoblots show the colocalization of rc-Maf and ubiquitin as well as K48. (F) HEK293 cells were treated with the procedures denoted below the immunoblots gels. Immunoblots show abundance of rc-Maf. The data are from one experiment that represents three independent experiments. Abbreviations: NC: naive control. AA: airway allergy. IP: immunoprecipitation. Ab: antibody.

The data of Figure 3 show the elevated *Trim41* expression in airway DCs, which was negatively correlated with the *Il10* expression in DCs (Figure S4). TRIM41 is a ubiquitin E3 ligase that can facilitate the degradation of targeting proteins.^19^ Our assumption was that TRIM41 could be involved in reducing Il10 expression in DCs. To test this, protein samples were extracted from purified DCs and analyzed by Western blotting. We observed reduced amounts of c-Maf, the transcription factor for the *Il10* gene, in AA DCs (Figure 4A). By immunoprecipitation (IP) with an anti-TRIM41 Ab as a bait, a complex of TRIM41 and c-Maf (a transcription factor of the *Il10* gene) was detected (Figure 4B). Ubiquitin and K48 were colocalized with c-Maf (Figure 4C). The findings indicate that the increase in TRIM41 could lead to a decrease in c-Maf in AA DCs. To verify this, HEK293 cells were transfected with His-*Trim41* plasmids (Figure S5) and Flag-*Maf* plasmids (Figure S6). Indeed, a complex of recombinant (r) TRIM41 and rc-Maf was found in the cells (Figure 4D). Colocalization of rc-Maf and ubiquitin and K48 was also detected (Figure 4E). The presence of TRIM41 induced c-Maf degradation in HEK293 cells, which could be inhibited by the presence of MG138 (a protease inhibitor) (Figure 4F). The results indicate that TRIM41 interferes with the expression of *Il10* in DCs by inducing c-Maf degradation.

### ANXA10 counteracts TRIM41 to promote the *Il10* expression in DCs

**Figure 5:**
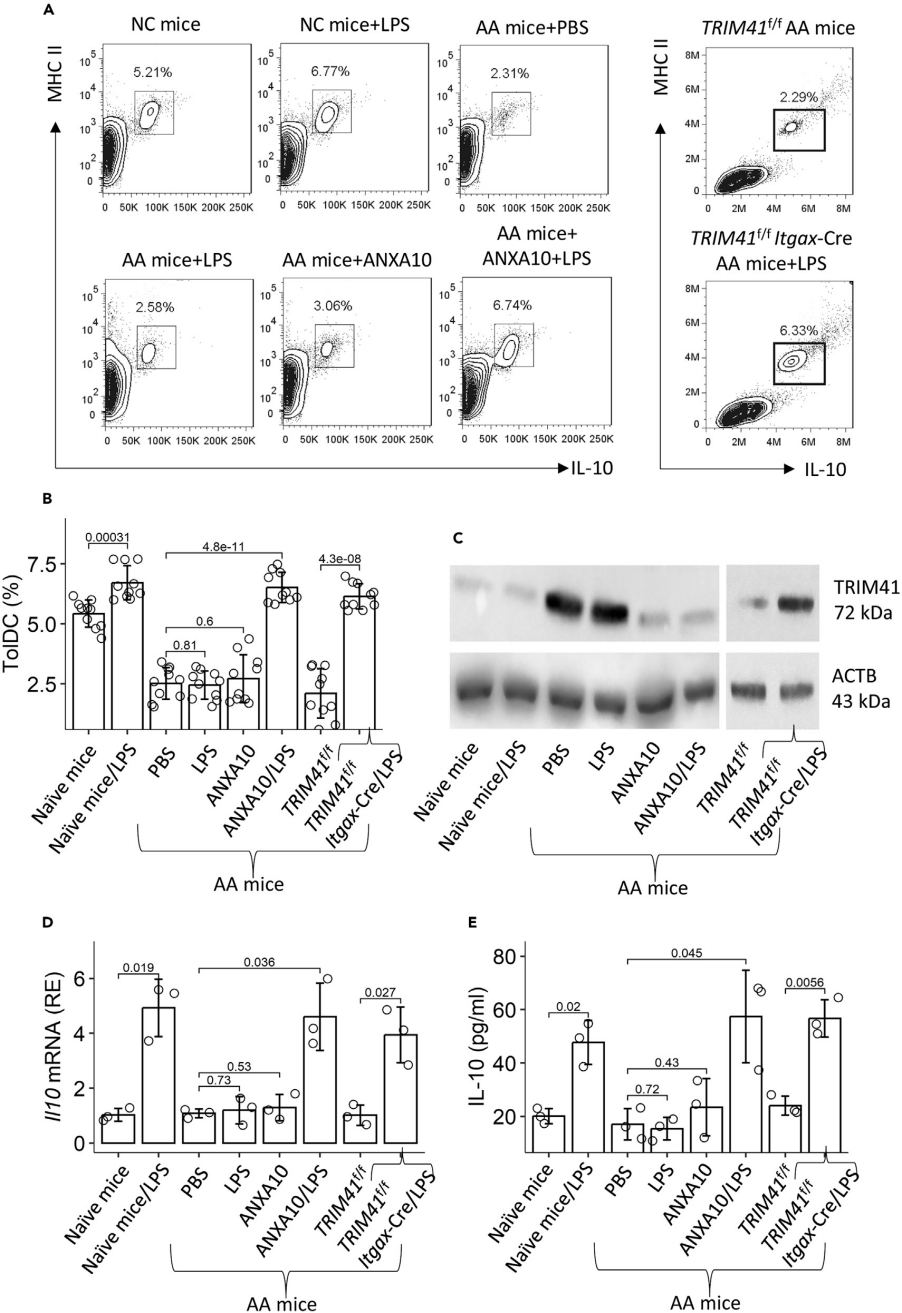
Inhibition of TRIM41 restores the *Il10* expression in AA airway DCs (A–C) Mice were treated with the procedures denoted above each FCM panel for one week. (A) AMCs were prepared and analyzed by FCM. Gated FCM plots show induced TolDCs in the airways (the DC gating strategy is presented in Figure S2). (B) Mean ± SD of TolDC counts from 10 mice per group. (C) DCs were purified from AMCs. Proteins were extracted from DCs and analyzed by western blotting. Immunoblots show the protein amounts of TRIM41 in DCs (the data are from one experiment that represents 3 independent experiments with pooled samples per group). (D and E) DCs were isolated from AMCs of NC mice and AA mice (denoted on the X axis). Recombinant ANXA10 (100 nMol) or/and LPS (1 μg/mL) was added to DC culture for 48 h. DCs were harvested to be analyzed by RT-qPCR; supernatant was analyzed by ELISA. The bars show mean ± SD of the *Il10* mRNA amounts in DCs (D) and IL-10 quantity in supernatant (E). Each dot in bars presents one sample (tested in triplicate). Statistics: ANOVA + Bonferroni test. *p* values are presented in figures. Each group consists of 10 mice. Abbreviations: NC: naive control. AA: airway allergy. AMC: airway mononuclear cell. *Trim41*^f/f^*Itgax*-Cre: mice carrying *Trim41*-deficient DCs (*Trim41*^f/f^ is the control mice).

We next tested the effects of annexin A10 (ANXA10), an inhibitor of TRIM41,^19^ on promoting the *Il10* expression in DCs. AA mice were treated with nasal instillations (containing ANXA10 or/and LPS) daily for 7 days. AMCs were prepared from the mice, and analyzed by FCM. We found that the frequency of IL-10^+^ DCs was markedly increased in AA mice treated with ANXA10 and LPS as compared with those treated with either ANXA10 or LPS alone (Figures 5A and 5B). Airway DCs were then isolated from AMCs of AA mice and analyzed by Western blotting. We found that the amounts of TRIM41 were higher in AA DCs as compared to NC DCs. Treatment with ANXA10 significantly reduced the amounts of TRIM41 in AA DCs (Figure 5C). On the other hand, the isolated DCs were exposed to ANXA10 or/and LPS in culture. We observed that LPS efficiently induced the *Il10* expression in NC DCs but not in AA DCs. The presence of ANXA10 and LPS significantly increased the *Il10* expression in AA DCs (Figure 5D). IL-10 was detected in culture supernatant, which was elevated in AA DC culture after exposure to both ANXA10 and LPS but not in either one alone (Figure 5E). According to the results, ANXA10 can restore the immune tolerogenic functions of DCs. The data are corroborated by using mice carrying the *Trim41* gene-deficient DCs in the experiments (Figure 5).

### Inhibition of TRIM41 mitigates AA response

**Figure 6:**
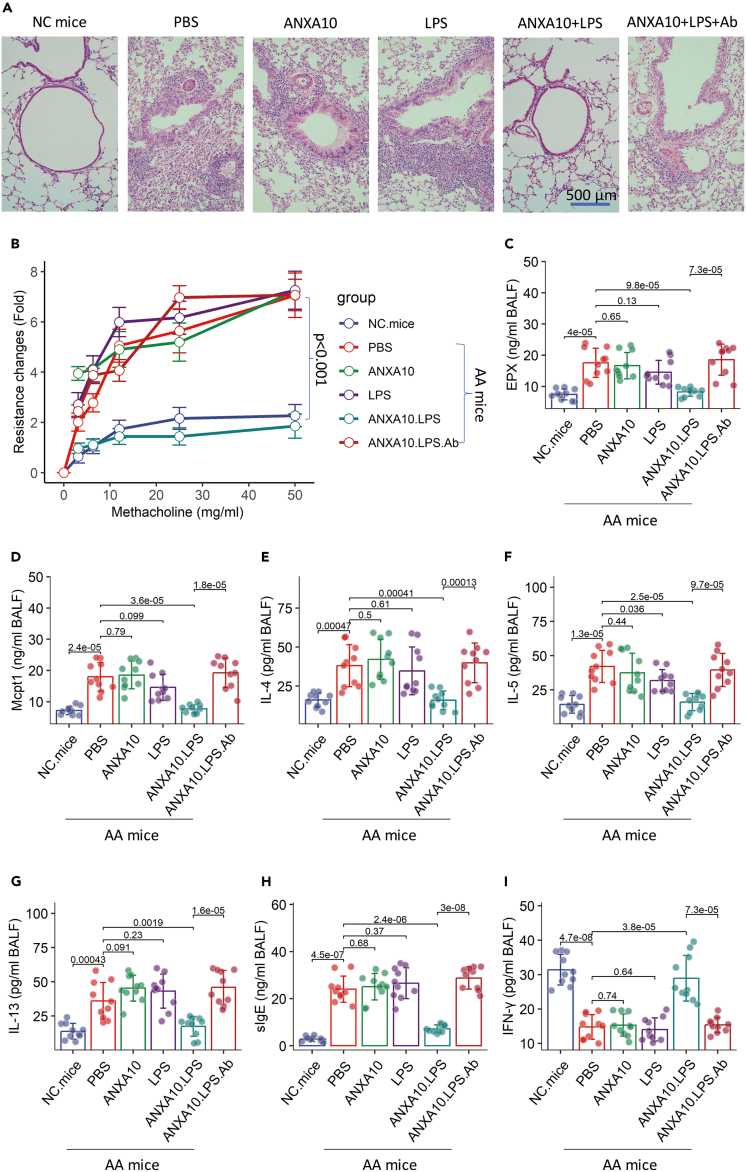
The ANXA10/LPS therapy suppresses AA response The treatment of AA mice is denoted above each histology panel. (A) Representative histology of the lung (original magnification: ×200). (B–I) Mean ± SD of the indicated molecule quantity in BALF. Each dot in bars presents one sample (tested in triplicate). Statistics: ANOVA + Bonferroni test. *p* values are presented in figures. Each group consists of 10 mice. Abbreviations: NC: naive control. AA: airway allergy. Ab: a neutralizing Ab of IL-10. ANXA10/LPS: mice received nasal instillations (20 μL/nostril; containing ANXA10 5 μM and LPS 20 μg/mL) daily for one week.

**Figure 7:**
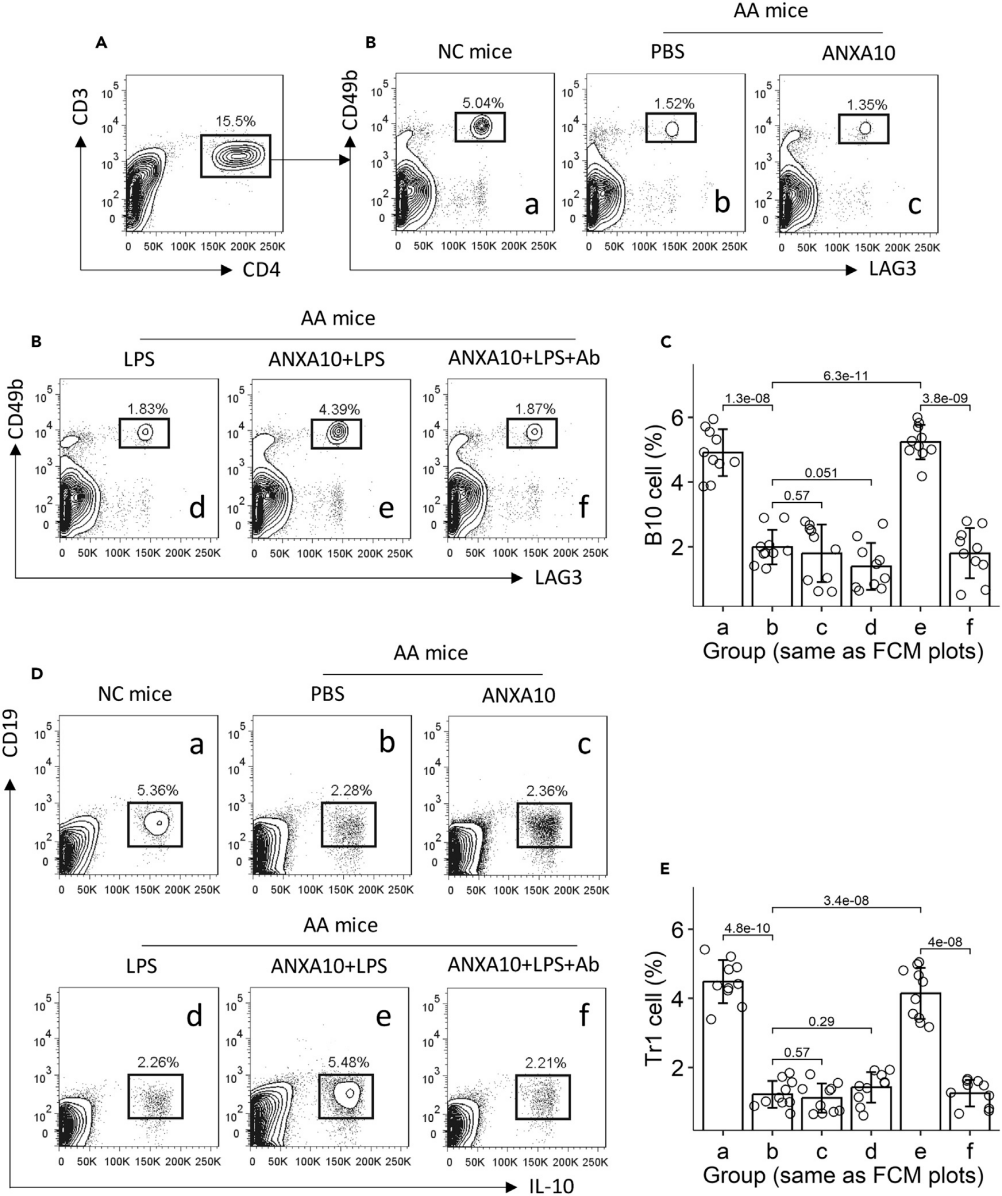
ANXN10/LPS therapy upregulates the number of Tr1 cells and B10 cells in the airways of AA mice AMCs were isolated from the airway tissues of mice as described in Figure 6. The AMCs were analyzed by FCM. (A) CD3^+^CD4^+^ T cells were gated, from which Tr1 cells were gated (B). (C) Mean ± SD of Tr1 cell counts from 10 mice per group. (D) Gated cells are B10 cell. (E) Mean ± SD of B10 cell counts from 10 mice per group. Each dot in bars presents one sample. Statistics: ANOVA + Bonferroni test. *p* values are presented in figures. Each group consists of 10 mice. Abbreviations: NC: naive control. AA: airway allergy. FCM: flow cytometry. Ab: a neutralizing Ab of IL-10.

An AA mouse model was established (Figure S1). Lung inflammation, airway hyperresponsiveness, elevated levels of allergic mediators, Th2 cytokines, and sIgE in BALF were observed in the mice as part of their AA responses. Mice were treated with nasal instillations containing ANXA10 and LPS daily for one week. The mice were challenged with specific antigens (DME) one day after the treatment. ANXA10 and LPS administration effectively suppressed the AA response according to the results. To test the role of IL-10 in the effects of ANXA10/LPS therapy, a neutralizing Ab was added to the nasal instillations of ANXA10/LPS to treat AA mice. As expected, the therapeutic effects were abolished (Figure 6). Additionally, we found that the ANXA10/LPS therapy significantly increased the frequency of B10 cells and Tr1 cells in the AMCs of AA mice (Figure 7). The results demonstrate that the ANXA10/LPS therapy can efficiently suppress AA response.

## Discussion

The present study revealed that TRIM41 interfered with the *Il10* expression by causing c-Maf degradation in airway DCs of AA mice. This compromised the immune tolerogenic functions of DCs. Inhibition of TRIM41 by ANXA10 suppressed experimental AA by restoring the immune tolerogenic functions in airway DCs.

The data show elevated amounts of TRIM41 in airway DCs of AA mice. The expression of *Trim41* in DCs was negatively correlated with the tolerogenic functions of DCs. DCs are the important immune cell fraction in the body. The initiation of immune response is dictated by DCs. Induction of immune tolerance is one of the important functions of DCs. Cumulative reports indicate that the dysfunctional tolerogenicity of DCs is associated with the pathogenesis of many immune diseases.^20^ Reduction of tolerogenic DCs in infant stage is associated with the development of AA.^21^ To increase tolerogenic DCs with Fms-related tyrosine kinase 3 ligand (Flt-3L) can alleviate experimental asthma in mice.^22^ However, factors causing the reduction of tolerogenic DCs remain unclear. Current data show that the elevated levels of TRIM41 in DCs are associated with the dysfunction of DC’s tolerogenic functions. This progress sheds light on further understanding the mechanism of tolerogenic DC dysfunction and related immune disorders.

The data show that the *Trim41* expression in DCs is positively correlated with the AA responses. It is known that the Th2 response plays a critical role in the pathogenesis of AA, while the causative factors for the initiation of Th2 polarization in the airways remain incompletely understood. Our previous studies show that DC-derived TIM4 (T cell immunoglobulin domain molecule-4) is a crucial factor in the initiation of Th2 responses.^23^^,^^24^ Type 2 DC-derived interferon regulatory factor 4 and Krüppel-like factor 4 can induce Th2 cell development.^25^ Current data reveal an important factor, TRIM41, associated with the pathogenesis of AA. TRIM41 is positively correlated with the Th2 cytokines in BALF, indicating that TRIM41 may contribute to the development of Th2 polarization.

It is known that immune responses are tightly regulated by the immune regulatory system in the body.^26^ B10 cells and Tr1 cells are the major cell fractions of immune regulatory cells. Both B10 cells and Tr1 cells produce IL-10, and they can be induced by tolerogenic DCs.^27^^,^^28^ Tolerogenic DCs play a critical role in the induction of immune regulatory cells.^29^ The number of B10 cells and Tr1 cells in the airway tissues of AA mice is lower than that of NC mice, as shown by our data. DCs isolated from NC mouse airway tissues are capable of inducing B10 cells and Tr1 cells, while DCs from AA mice induced significantly fewer B10 cells and Tr1 cells. This phenomenon is associated with the expression of *Trim41* in DCs, suggesting a link between DC’s immune tolerogenic properties and the expression of Trim41. The negative regulatory role of TRIM41 in DC’s tolerogenic functions can be one of the factors inducing Th2 polarization. This needs to be further investigated.

We found low levels of *Il10* expression in airway DCs of AA mice. There are several tolerogenic molecules produced by tolerogenic DCs, such as IL-10, transforming growth factor (TGF)-β, and IL-35.^30^ It is recognized that IL-10 plays a critical role in maintaining the homeostasis in the body.^31^^,^^32^ Mice with IL-10 deficiency can spontaneously suffer from immune disorders.^33^^,^^34^ Thus, the IL-10-producing tolerogenic DCs have attracted more attention.^35^ Current data show that airway DCs of AA mice exhibit impaired immune tolerogenic ability during the induction of B10 cells and Tr1 cells. Such a defect is associated with the expression of *Trim41* in DCs. TRIM41 is a ubiquitin E3 ligase and has the ability to facilitate targeting protein degradation through the ubiquitination mechanism.^19^ Such a phenomenon also occurred in our experimental system. The IP experiments revealed a complex of TRIM41 and c-Maf, where c-Maf and ubiquitin were found to be colocalized. The induction of c-Maf degradation by TRIM41 was verified by an *in vitro* experiment. Thus, the scenario is that in an allergic environment, DCs express high levels of *Trim41*, which induces c-Maf degradation. And thus, the expression of *Il10* is suppressed since c-Maf is the transcription factor of the *Il10* gene.

The data indicate that TRIM41 impairs the expression of Il10 in DCs, leading to suppression of DCs’ immune tolerogenic functions. Logically, inhibiting TRIM41 may promote or restore the immune tolerogenic condition in the airways. Disruption of the immune tolerance is one of the key factors in the pathogenesis of allergic disorders.^36^^,^^37^ Our data show that there are fewer B10 cells and Tr1 cells in the airway tissues of AA mice. B10 cells and Tr1 cells are the important components of the immune tolerant system.^38^^,^^39^ The fact indicates that the immune tolerance is disrupted in the airways of AA mice. The disruption of immune tolerance could be due to the ineffective induction of B10 cells and Tr1 cells by AA airway DCs. The treatment with the TRIM41 inhibitor alleviated AA responses verifies this inference. The treatment resulted in the recovery of B10 cells and Tr1 cells in the airway tissues. TRIM41 inhibition can restore the immune tolerant system in the airways, as evidenced by current data.

It is widely known that DCs can be subdivided into multiple subtypes, including conventional type 1 DCs (DC1), DC2, and plasmacytoid DCs (pDCs).^40^ DC1 induces a Th1 response, DC2 induces a Th2 response, and pDCs produce IFN-γ and play an important role in the anti-viral action.^41^^,^^42^ It appears that DCs’ IL-10 production is influenced by proper stimuli and environment. For example, exposure to LPS, the major components of bacterial cell walls, can induce the production of IL-10 by DCs.^43^^,^^44^^,^^45^ Thus, the present study analyzed the association of whole-DC activities with the immune tolerogenic status in the airways. This experimental setting generally reflects the actual condition in the airways.

In summary, the present data have revealed elevated *Trim41* expression in airway DCs of AA mice. TRIM41 can interfere with the expression of *Il10* in DCs by inducing c-Maf ubiquitination and degradation. Thus, TRIM41 plays an important role in the induction of immune tolerance disruption in the airways, which may be a critical factor in the pathogenesis of AA. The inhibition of TRIM41 can alleviate experimental AA that has the potential to be translated into a novel therapy for AA.

### Limitations of the study

All the data of this paper are generated from animal model studies, which may still be some differences from human samples. Further studies using human samples are warranted. On the other hand, IL-10 is also a canonical immune regulatory cytokine in other immune regulatory cells, such as Tr1 cells. Whether TRIM4 also compromises the immune regulatory ability of Tr1 cells may be further investigated.

## STAR★Methods

### Key resources table

**Table:** 

REAGENT or RESOURCE	SOURCE	IDENTIFIER
**Antibodies**		

CD14	Santa Cruz Biotech	sc-515785, AF488
CD3	Santa Cruz Biotech	sc-20047, AF546
CD19	Santa Cruz Biotech	sc-390244, AF594
LAG3	Santa Cruz Biotech	sc-32750, AF647
CD49b	Santa Cruz Biotech	sc-74466, AF680
B220	Santa Cruz Biotech	sc-19597, AF680
CD62L	Santa Cruz Biotech	sc-390756, AF488
IL-10	Santa Cruz Biotech	sc-365858, AF680
MHC II	Santa Cruz Biotech	sc-13556, AF700
F4/80	Santa Cruz Biotech	sc-365340, AF790
c-Maf	Santa Cruz Biotech	518062
Dust mite extracts	Dakewe BioMart	NA
TRIM41 Ab	Dakewe BioMart	59-164
K48 Ab	Dakewe BioMart	M02848-1

**Critical commercial assays**		

EPX ELISA kit	Dakewe BioMart	NA
Mite specific IgE ELISA kit	Dakewe BioMart	NA
IL-4 ELISA kit	Dakewe BioMart	NA
IL-5 ELISA kit	Dakewe BioMart	NA
IL-13 ELISA kit	Dakewe BioMart	NA

**Chemicals, peptides, and recombinant proteins**		

ANXA10	Sangon Biotech	NA

**Deposited data**		

RNA sequencing dataset	NCBI	PRJNA1112352

**Software and algorithms**		

R v4.3.2	R Development Core Team, 2022	https://mirrors.bfsu.edu.cn/CRAN/
FlowJo v10	BD	https://www.flowjo.com/
BD FACSCanto II Clinical Software	BD	https://www.bdbiosciences.com/en-eu/products/software/instrument-software/bdfacscanto-clinical-software

### Resource availability

#### Lead contact

Further information and requests for resources and reagents should be directed to and will be fulfilled by the lead contact: Pingchang Yang (pcy2356@163.com).

#### Materials availability

All new reagents are available in our lab for non-commercial research.

#### Data and code availability

This paper includes all the data. We do not have specific code for this paper. Any additional information required to reanalyze the data reported in this paper is available from the lead contact upon request.

### Experimental model and study participant details

#### Ethics statement

The experimental protocol was reviewed and approved by the animal ethics committee at our institution. All animal experiments were conducted in accordance to the ARRIVE guidelines.

### Method details

#### Mice

Male BALB/c mice with an age range of 6-8 weeks were procured from Guangdong Experimental Animal Center in Fushan, China. Mice were maintained in a specific pathogen-free facility at our institution, with free access to water and food. The Animal Ethics Committee at our institution gave its approval to the animal experimental protocol. The ARRIVE guidelines were adhered to during the experiments.

#### Establishment of a murine model of airway allergy (AA)

Following published procedures,^24^ randomly grouped mice were subcutaneously injected with dust mite extracts (DME, 0.1 mg per mouse in 0.1 ml alum) on day 1 and day 7, respectively. Mice received nasal instillations (20 μl/nostril containing 5 mg DME/ml PBS) daily from day 9 to day 22. Mice received a large dose of antigen challenge using nasal instillations (20 μl/nostril containing 50 mg DME/ml PBS) on day 23 or after treatment. The AA responses were evaluated afterward (as shown below).

#### Assessment of the airway response

Airway hyperresponsiveness to methacholine was recorded using the unrestrained whole-body plethysmography following published procedures.^46^ The mice inhaled methacholine in escalating doses (0, 6.25, 12.5, 25, 50, 100 mg/mL). The Penh value was recorded.

#### AA responses assessment

After receiving a large dose of antigen challenge, the mice were sacrificed by cervical dislocation. The trachea was opened in the neck. Saline (1 ml/mouse) was introduced into the airways using a syringe. Instantly, the saline was retrieved and used as bronchoalveolar lavage fluid (BALF). The AA responses were determined by ELISA analysis of BALF. The amounts of eosinophil peroxidase (EPX), mouse mast cell protease-1 (Mcpt1), IL-4, IL-5, IL-13, and mite specific IgE in BALF were used as indicators of AA responses.

#### Enzyme-linked immunosorbent assay (ELISA)

ELISA was used to determine cytokines and IgE in BALF and culture supernatant, following the manufacturer's instructions.

#### Lung histology

A piece of the lung tissue was excised upon the sacrifice. Paraffin sections were prepared from the lung tissue, stained with hematoxylin and eosin, and observed using a light microscope. Photomicrographs of interested area were taken at ×200 magnification.

#### Preparation of single airway granular cells (AGCs)

After the sacrifice, the lung fragments were removed, chopped, and incubated with collagenase IV (0.5 mg/ml) and DNase I (200 ng/ml) for 30 minutes at 37°C with moderate agitation. A cell strainer was used to filter single cells (70 μm first, then 40 μm). AGCs were separated from the single cells by the gradient density Percoll centrifugation.

#### Cell culture

RPMI 1640 medium was used to culture cells. 10 % fetal calf serum, 100 U/ml penicillin, 0.1 mg/ml streptomycin, and 2 mM L-glutamine were added to the medium. The Trypan blue exclusion assay was used to assess cell viability, and it exceeded 99%.

#### Flow cytometry (FCM)

In the surface staining, cells were stained with fluorescence labeled Abs of interest (1 μg/ml) or isotype IgG for 30 min at 4°C. Cells were washed with phosphate-buffered saline (PBS, containing 2% bovine serum albumin), and analyzed with a flow cytometer (BD FACSCanto II). To stain the intracellular molecules, cells were exposed to brefeldin A (10 μg/ml) for 4 h, and fixed with 1% paraformaldehyde (containing 0.05% Triton X-100) for 1 h. After washing with PBS, the cells were processed using surface staining procedures. The data were analyzed using a software package, Flowjo (TreeStar Inc., Ashland, OR) with the data obtained from the isotype IgG staining as gating references.

#### Isolation of immune cells

Single cells were stained with fluorescence-labeled Abs (detailed in figures), and isolated by FCM cell sorting. To isolate DCs from AGCs, the CD19^+^, CD14^+^, and F4/80^+^ cells were gated out first. MHC II^+^ cells were sorted from the remaining cells to be used as DCs in other experiments. B220^+^ CD19^+^ B cells and CD3^+^CD4^+^CD62L^+^ T cells were isolated from the mouse spleen cells by FCM cell sorting. Cell purity of isolated cells was checked by FCM. If it did not reach 90% or over, cells were sorted again.

#### RNA sequencing (RNAseq)

DCs were isolated from AGCs and sent to a biotech company (Shenzhen BGI, China) for analysis by RNAseq. The data were analyzed by the technical staff of the company. The differentially expressed genes (DEGs) were enriched using the software DESeq2 and presented in a heatmap.

#### Real-time quantitative RT-PCR (RT-qPCR)

Cells collected from relevant experiments were used to extract RNA samples and convert them to cDNA using a reverse transcription kit. A SYBR Green Master Mix kit was used to amplify the cDNA samples in a qPCR device (Bio Rad CFX96) with relevant primers. Primers used in the present study include *Cmip* (ctgctgtccgactacgatga, cagggctgtagagctggaac), *Maf* (aaggaggaggtgatccgact, tctcctgcttgaggtggtct), *Stat3* (gacccgccaacaaattaaga, tcgtggtaaactggacacca), Il10 (ccaagccttatcggaaatga, ttttcacaggggagaaatcg), Bst2 (caatctacttcgccgtcaca, tcttctccagggactcctga), *Cd45r* (cctgctcctcaaacttcgac, gacacctctgtcgccttagc), *Cd80* (ccatgtccaaggctcattct, ttcccagcaatgacagacag), *Cd83* (gcctccagctcctgtttcta, ttggatcgtcagggaatagg), *Cd86* (cacgagctttgacaggaaca, ttaggtttcgggtgaccttg), *Cd172a* (gcttctctccccggaatatc, caaggtgatgtgggctacct), *Cd11c* (tgatgagccagcttcagaga, tagccgaggctgtgtatgtg), *Trim41* (cggctactgcaagacatcaa, gtcagggtccagtgtcaggt). The data were processed with the formula of 2^-ΔΔCt^, and presented as relative expression against a housekeeping gene *Actb*.

#### Western blotting

Protein samples were extracted from cells collected from relevant experiments, fractionated by SDS-PAGE (sodium dodecyl sulfate-polyacrylamide gel electrophoresis) and transferred onto a polyvinylidene fluoride membrane. The primary Abs (Ab types are detailed in figures. Abs were diluted to 200 ng/ml and used to incubate the membrane overnight, followed by incubating with second Abs (labeled with horseradish peroxidase, diluted to 20 ng/ml) for 2 h. After incubation, the membranes were rinsed with TBST (Tris-buffered saline containing 0.05% Tween 20). Enhanced chemiluminescence was used to develop immunoblots on the membrane and they were photographed using an imaging device. To colocalize c-Maf and ubiquitin or K48, after the staining procedure to illustrate c-Maf, the membranes were treated with a peeling buffer to remove the anti-c-Maf Ab, and followed by staining with ubiquitin Ab or K48 Ab.

#### Immunoprecipitation (IP)

Proteins were precleared by incubating the proteins with protein G agarose beads for 2 h. Centrifugation was used to remove the beads. The Abs of interest (Ab types are depicted in the figures) were incubated with samples overnight. Protein G agarose beads were included in the samples to capture the immune complex. Proteins on the beads were eluted using an eluting buffer, and analyzed by Western blotting.

#### Assessment of the binding between c-Maf and TRIM41

Plasmids of *Maf*-expression and *Trim41*-expression were provided by Sangon Biotech (Shanghai, China). HEK293 cells were transfected with the *Maf*-plasmids and *Trim41*-plasmids the reagent kit and protocol provided by the manufacturer. The effects of transfection (the induction of recombinant c-Maf and TRIM41) were checked by Western blotting 48 h after.

#### Assessment of restoring the *Il10* expression in airway DCs of AA mice by inhibiting TRIM41

DCs were isolated from the mouse airway tissues by FCM cell sorting. Lipopolysaccharide (LPS, 5 μg/ml) and recombinant annexin A10 (ANXA10, 100 nMol; provided by Sangon Biotech) were added to culture. Three days later, cells were harvested, and analyzed by FCM, RT-qPCR, and Western blotting to assess the production of IL-10 in DCs.

#### Inhibition of TRIM41 in AA mice

A mouse model of AA was established. One day after the completion of sensitization, mice received nasal instillations (20 μl/nostril; containing ANXA10 5 μM and LPS 20 μg/ml) daily for one week.

### Quantification and statistical analysis

Student's *t*-test was used to determine the difference between two groups. Multiple comparisons were conducted by performing an ANOVA followed by a Bonferroni test. The correlation between groups was determined by conducting a Pearson correlation coefficient test. p<0.05 was set as a significant criterion.
