# Supplementary material for: TRIM41 contributes to the pathogenesis of airway allergy by compromising dendritic cells’ tolerogenic properties
Source: iScience. 2024 May 21;27(6):110067. doi: 10.1016/j.isci.2024.110067 (PMC11176661; doi:10.1016/j.isci.2024.110067)
Supplement: Document S1. Figures S1–S6 [file mmc1.pdf]

iScience, Volume *27*

Supplemental information

TRIM41 contributes to the pathogenesis of airway allergy by compromising dendritic cells' tolerogenic properties

Qiuying Peng, Xiangqian Luo, Lihua Mo, Xuejie Xu, Yu Liu, Dabo Liu, and Pingchang Yang

Supplemental materials

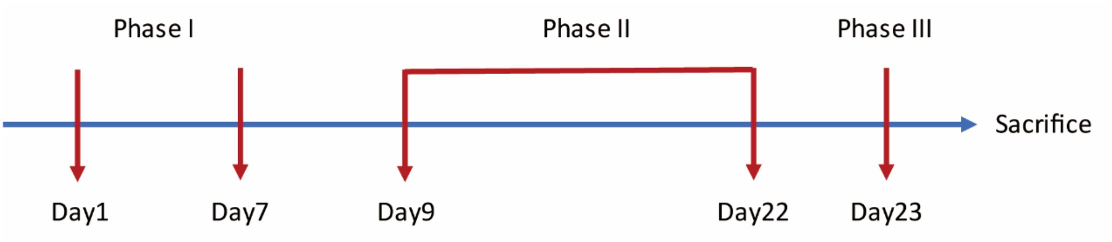

Figure S1. A schematic of AA establishment protocol. Related to Figure 1. Phase I: randomly grouped mice (10 mice per group) were subcutaneously injected with DME (0.1 mg/mouse in 0.1 ml alum) on day 1 and day 7, respectively. Phase II: Mice received nasal instillations (20 µl/nostril containing 5 mg DME/ml) daily from day 9 to day 22. Phase III: Mice received nasal instillations (20 µl/nostril containing 50 mg DME/ml).

Abbreviations: AA: Airway allergy. DME: Dust mite extracts.

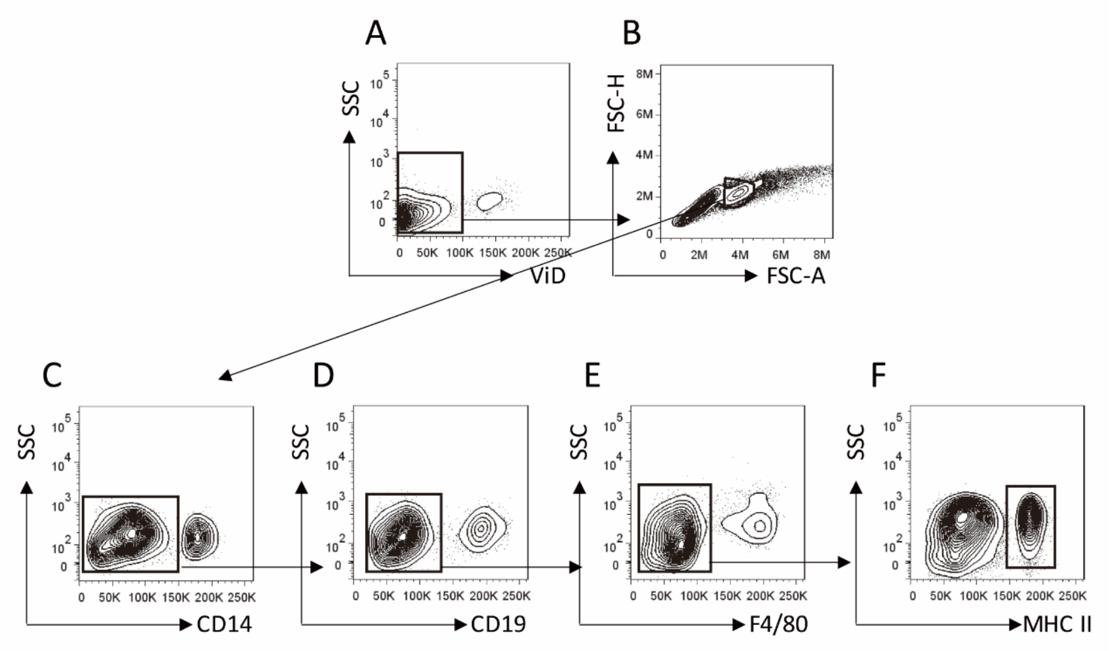

Figure S2. Gating strategy of DCs. Related to Figure 2. AMCs were prepared with the mouse airway tissues, and analyzed by FCM. A, dead cells were gated out. B, adherent cells were gated out. C, $CD14^+$ cells were gated out. D, B cells were gated out. E, macrophages were gated out. F, DCs were gated. Statistics: Student's *t*-test. *p* values are presented in figures.

Abbreviations: DC: Dendritic cell. AMC: Airway mononuclear cell. FCM: Flow cytometry. ViD: An amine reactive fluorescence dye using to stain dead cells.

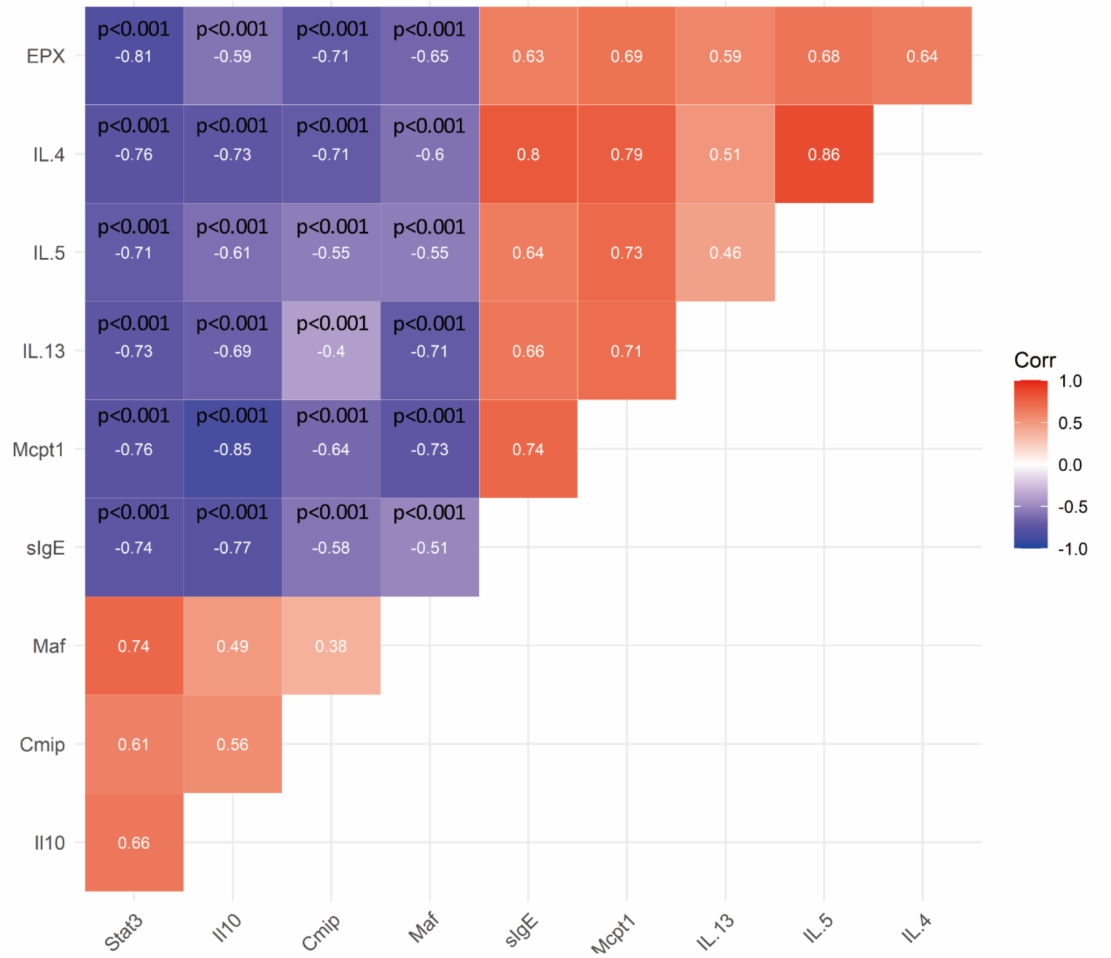

Figure S3. Correlation between *Il10* expression related genes in airway DCs and AA response. Related to Figure 3. Correlation test was performed with the data of AA response (presented in Fig. 2) and Il10 expression related genes (presented in Fig. 3). A heatmap shows the correlation coefficients. Statistics: Pearson correlation coefficient test. *p* values are presented in the heatmap.

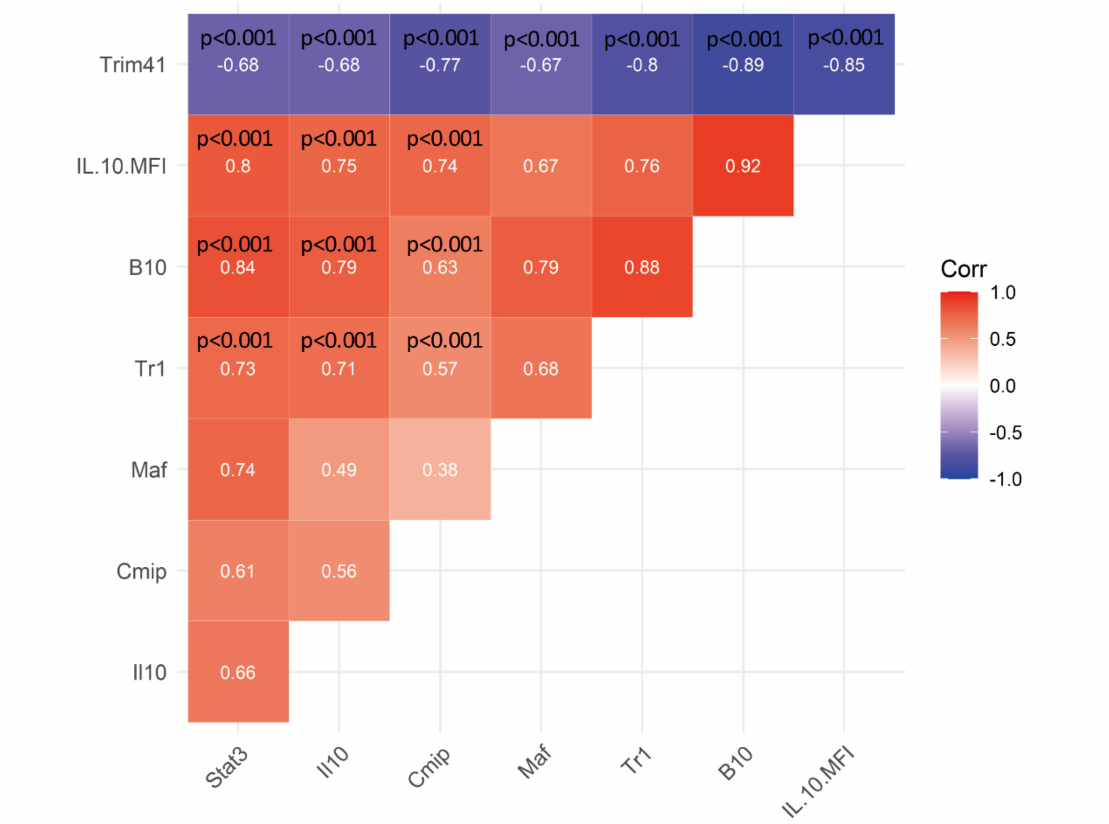

Figure S4. *Il10* expression in DCs are correlated with DC's tolerogenic functions. Related to Figure 3. Correlation test was performed with the data of DC tolerogenic functions (the induction of B10 cells and Tr1 cells; data are presented in Fig. 1) and *Il10* expression related genes (presented in Fig. 3). A heatmap shows the correlation coefficients. Statistics: Pearson correlation coefficient test. *p* values are presented in the heatmap.

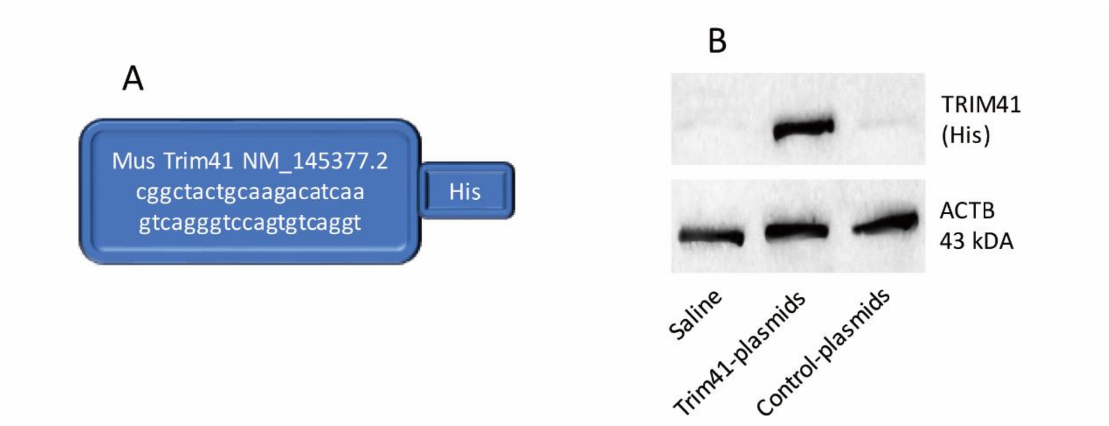

Figure S5. Information of recombinant TRIM41 protein production. Related to Figure 4. A, gene information of mouse *Trim41*. B, recombinant TRIM41 produced by HEK293 cells.

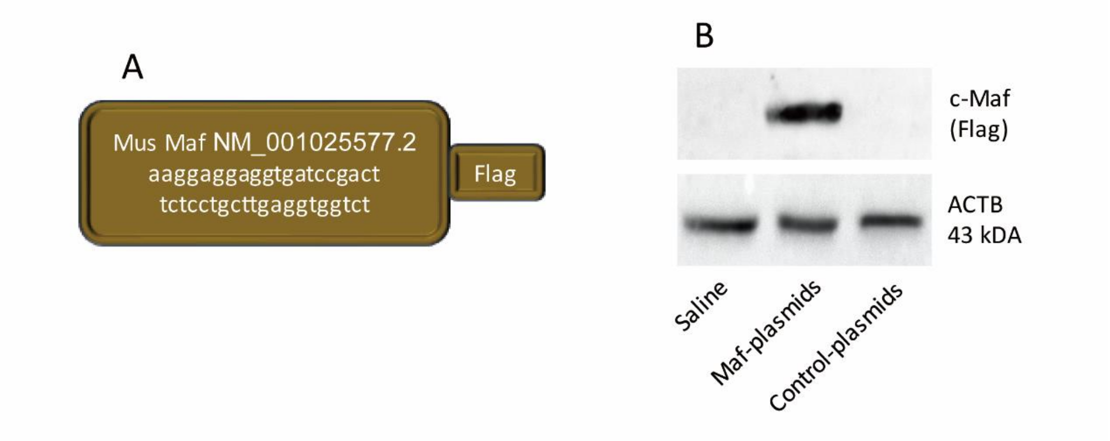

Figure S6. Information of recombinant c-Maf protein production. Related to Figure 4. A, gene information of mouse *Maf*. B, recombinant c-Maf produced by HEK293 cells.
